# Revisited – the species of Tweeting vineyard snails, genus *Cantareus* Risso, 1826 (Stylommatophora, Helicidae, Helicinae, Otalini)

**DOI:** 10.3897/zookeys.876.36472

**Published:** 2019-09-18

**Authors:** Houria Bouaziz-Yahiatene, Thomas Inäbnit, Ferroudja Medjdoub-Bensaad, Maria Stella Colomba, Ignazio Sparacio, Armando Gregorini, Fabio Liberto, Eike Neubert

**Affiliations:** 1 Laboratoire de Production, sauvegarde des espèces menacées et des récoltes, Influence des variations climatiques, Département de Biologie, Faculté des Sciences Biologiques et des Sciences Agronomiques, Université Mouloud Mammeri de Tizi-Ouzou, 15000, Algeria; 2 Natural History Museum of the Burgergemeinde Bern, Bernastrasse 15, CH-3005 Bern, Switzerland; 3 Institute of Ecology and Evolution, University of Bern, 3012 Bern, Switzerland; 4 University of Urbino, Dept. of Biomolecular Sciences, Via I. Maggetti 22, 61029 Urbino (PU), Italy; 5 Via Principe di Paternò 3, 90143 Palermo, Italy; 6 Via del Giubileo Magno n 93, 90015 Cefalù (PA), Italy

**Keywords:** Algeria, Italy, cryptic species, genetic characterisation, Algérie, Italie, Cantareus, espèce cryptique, caractérisation génétique

## Abstract

The generic allocation of *Helix subaperta* is clarified by using genetic data and morphological traits of the genital organs; its position within the hitherto monotypic genus *Cantareus* is corroborated. Further analysis of several specimens of *Cantareus apertus* from Algeria and Italy revealed that this taxon is composed of two species, *C. apertus* from Italy, and *C. koraegaelius* from Algeria. The morphological traits of the genital organs of all three species are discussed, and the definition of the genus *Cantareus* is amended. All three species confined to *Cantareus* are re-described, and the syntype specimen of *H. aperta* is illustrated.

## Abstract (fr)

La répartition générique de *Helix subaperta* est clarifiée en utilisant des données génétiques et des traits morphologiques des organes génitaux sa position au sein du genre *Cantareus* jusque-là monotypique est renforcée. Une analyse plus approfondie de plusieurs spécimens de *Cantareus apertus* d'Algérie et d'Italie arévélé que ce taxon est composé de deux espèces *C. apertus* d’Italie et *C. koraegaelius* d’Algérie. Les traits morphologiques des organes génitaux des trois espèces sont étudiés et la définition du genre *Cantareus* est modifiée. Les trois espèces confinées à *Cantareus* sont à nouveau décrites et le spécimen de syntype de *H. aperta* est illustré.

## Introduction

The hitherto monotypic genus *Cantareus* is currently placed in the helicoid tribe Otalini G. Pfeffer, 1930 (http://www.molluscabase.org/aphia.php?p=taxdetails&id=994951) (Neiber and Hausdorf 2015; Razkin et al. 2015). This clade embraces 12 genera of helicoid snails, among them *Cornu* Born, 1778 and *Erctella* Monterosato, 1894. Both genera contain species possessing shells that resemble each other to some extent, and they exhibit a similar morphology of their genital organs. The Sicilian genus *Erctella* was previously reviewed and re-described by Colomba et al. (2011), and resulted in the resurrection of three valid, narrowly endemic species of the group on north-western Sicily.

The tribe originates from the Maghrebinian radiation centre (Korábek et al. 2019), and contains a considerable number of species, many of them only randomly known. One of these problematic species is the enigmatic *Helix subaperta* Ancey, 1893, which is endemic for a relatively small mountain ridge in the Kabylie in eastern Algeria, the Djudjura Mountains. It has a shell that shows character states typical for both aforementioned genera: it resembles *Cornu* in its colouration but shows no malleate pattern, and *Erctella* in the shell form and the considerably developed ribs on the surface of the adult shell. Thus, the starting point for this paper was to clarify the correct taxonomic position of this species. However, adding supplementary specimens, and using genetic data available from other studies revealed that there is another and completely overlooked species living in northern Africa, which turns out to be a member of *Cantareus*, the Tweeting vineyard snail.

## Materials and methods

### Taxon sampling

The specimens for this study were collected by the authors of the study, particularly by the senior author. Missing sequences for *Erctella* and Italian *Cornu* were added for the same specimens used by Colomba et al. (2011; 2015). Freshly sampled animals were preserved in 80 % EtOH. The analysed specimens were removed from their shells, the genital organs were isolated and fixed on a wax-bed. The situs as well as the details of the interior lumina were photographed. Tissue samples were taken from those specimens and sequenced, the shells were photographed (in case they were not destroyed when extracting the animal). All shell photos were taken using a Leica M205 C microscope with the Leica DFC425 camera and the IMS Client (Imagic Bildverarbeitungs AG, Glattbrugg, Switzerland).

### Acronyms

**ANSP** Academy of Natural Sciences, Philadelphia, USA

**NHMW** Natural History Museum Vienna, Austria

**NMBE** Natural History Museum Bern, Switzerland

**SMF** Senckenberg Research Institute Frankfurt am Main, Germany

### Abbreviations

**H** shell height

**D** shell diameter

**PH** peristome height

**PD** peristome diameter

### DNA Extraction, PCR amplification, and sequencing

#### Phylogenetic analysis

DNA was extracted from a piece of foot muscle tissue using Qiagen Blood and Tissue Kit (Qiagen cat nr. 69506) and the QIAcube extraction robot (Protocol 430, DNeasy Blood Tissue and Rodent tails Standard). Our phylogenetic hypotheses were reconstructed using five phylogenetic markers (mitochondrial COI (657 base pairs (bp)), 16S (374 bp) and nuclear 28S (528 bp), H3 (304 bp) and ITS2 (909 bp)), resulting in a length of 2772 bp (see Table 1).

**Table 1.** Taxa used in this study: family, species, locality, voucher, GenBank accession numbers for COI, 16S, H3, and 5.8S-ITS2-28S.

Species	Locality	Coordinates		Voucher	GenBank accession number					
					CO1	16S	H3	28S	5.8S-ITS2	Origin
		(N)	(E)							
*Helix pomatia*	Hannover-Anderten, N side of Mittelland Canal/ Lower Saxony	52.3586, 9.8681		MN_2551-Hel/MN_012	KR705053	KR705016	KR705127	KR705116	KR705093	Neiber and Hausdorf (2015)
*Massylaea vermiculata*	Makouda, Tizi Ouzou, DZ	36.7909, 4.0659		NMBE 540544	MF564159	MF564112	MF564174	MF564128	MF564144	Bouaziz-Yahiatene et al. (2017)
	Beach between Agia Napa and Capo Greco, CY	34.9728, 34.0427		NMBE 519919	MF564160	MF564113	MF564175	MF564129	MF564145	Bouaziz-Yahiatene et al. (2017)
*Massylaea constantina*	Draâ-Ben Khedda/ Tizi Ouzou, DZ	36.7318, 3.9654		NMBE 534211_1	MF564164	MF564118	MF564181	MF564134	MF564150	Bouaziz-Yahiatene et al. (2017)
	Draâ-Ben Khedda/ Tizi Ouzou, DZ	36.7318, 3.9654		NMBE 534211_2	MF564165	MF564119	MF564182	MF564135	MF564151	Bouaziz-Yahiatene et al. (2017)
*Cantareus subapertus*	Ighil Bourmi, DZ	36.4872, 4.0613		NMBE 550458_1	MK883426	MK883301	MK883382	MK883375	MK883376	This work
	Ighil Bourmi, DZ	36.4872, 4.0613		NMBE 550458_2	MK883427	MK883302	MK883383	MK883335	MK883377	This work
*Cantareus koraegaelius*	Tigzirt/ Tizi Ouzou, DZ	36.8901, 4.1279		NMBE 534199	MK883424	MK883294	MK883384	MK883336	MK883378	This work
	Draa Ben Kheda/ Tizi Ouzou, DZ	36.7318, 3.9654		NMBE 519923	MK883425	MK883295	MK883385	MK883337	MK883379	This work
	Djelfa, Algeria	34.6704, 3.2504		MVHN-2013	-	KJ458491	-	-	KJ458589	Razkin et al. (2015)
*Cantareus apertus*	Marincola, Amantea, Calabria	39.1128, 16.0797		NMBE 560941_1	MK883423	MK883300	MK883388	MK883338	MK883380	This work
	Marincola, Amantea, Calabria	39.1128, 16.0797		NMBE 560941_2	MK883422	MK883296	MK883389	MK883339	MK883381	This work
	Palermo: Cefalú, Cocuzzola	38.0247, 13.9417			KR921883	MK883297	MK883412	MK883345	GQ402427	Colomba et al. (2011, 2015, this work)
	Enna: Assoro, C. da Cernigliere	37.6331, 14.4075			KR921884	MK883298	MK883413	MK883348	GQ402428	Colomba et al. (2011, 2015, this work)
	Enna: Assoro, C. da Cernigliere	37.6331, 14.4075			KR921885	MK883299	MK883414	MK883368	GQ402429	Colomba et al. (2011, 2015, this work)
	Italy, Strada del Casone (Siena)	43.2363, 11.4631		FGC 36599	KU869798	KU870009	-	-	-	Fiorentino et al. (2016)
	Italy, Strada del Casone (Siena)	43.2363, 11.4631		FGC 36599	KU869799	KU870008	-	-	-	Fiorentino et al. (2016)
	Italy, Strada del Casone (Siena)	43.2363, 11.4631		FGC 36599	KU869800	KU870006	-	-	-	Fiorentino et al. (2016)
*Cornu aspersum*	Draa Ben Kheda/ Tizi Ouzou, DZ	36.7318, 3.9654		NMBE 519921	MK883429	MK883304	MK883387	MK883341	-	This work
	Ait Bouadou, Tizi Ouzou, DZ	36.5036, 4.0546		NMBE 534201	MK883428	MK883303	MK883386	MK883340	-	This work
	Palermo: Cefalú, Mazzaforno	38.0267, 13.9669			KR921888	MK883305	MK883392	MK883342	GQ402424	Colomba et al. (2011, 2015, this work)
	Palermo: Cefalú, Mazzaforno	38.0267, 13.9669			KR921887	MK883307	MK883391	MK883343	GQ402425	Colomba et al. (2011, 2015, this work)
	Palermo: Cefalú, Mazzaforno	38.0267, 13.9669			KR921886	MK883306	MK883390	MK883344	GQ402426	Colomba et al. (2011, 2015, this work)
*Erctella insolida*	Trapani: San Vito lo Capo, Cala Mancina	38.1786, 12.7186			KR921898	MK883332	MK883403	MK883363	GQ402457	Colomba et al. (2011, 2015, this work)
	Trapani: San Vito lo Capo, Cala Mancina	38.1786, 12.7186			KR921899	MK883333	MK883404	MK883355	GQ402458	Colomba et al. (2011, 2015, this work)
	Trapani: San Vito lo Capo, Cala Mancina	38.1786, 12.7186			KR921900	MK883334	MK883405	MK883356	GQ402459	Colomba et al. (2011, 2015, this work)
	Trapani: Custonaci, Monte Cofano	38.1075, 12.6831			KR921896	MK883331	MK883399	MK883346	GQ402447	Colomba et al. (2011, 2015, this work)
	Trapani: Custonaci, Monte Cofano	38.1075, 12.6831			KR921897	MK883330	MK883400	MK883347	GQ402448	Colomba et al. (2011, 2015, this work)
	Trapani: Custonaci, Monte Cofano	38.105, 12.6725			-	MK883327	MK883408	MK883349	GQ402440	Colomba et al. (2011, 2015, this work)
	Trapani: Custonaci, Monte Cofano	38.105, 12.6725			KR921893	MK883326	MK883409	MK883350	GQ402441	Colomba et al. (2011, 2015, this work)
	Trapani: Custonaci, Monte Cofano	38.105, 12.6725			KR921894	MK883328	MK883396	MK883351	GQ402442	Colomba et al. (2011, 2015, this work)
	Trapani: Custonaci, Monte Cofano	38.105, 12.6725			KR921895	MK883329	-	MK883352	GQ402443	Colomba et al. (2011, 2015, this work)
*Erctella cephalaeditana*	Palermo: Cefalú, La Rocca	38.0389, 14.0264			KR921889	MK883308	MK883393	MK883357	GQ402430	Colomba et al. (2011, 2015, this work)
	Palermo: Cefalú, La Rocca	38.0389, 14.0264			KR921890	MK883309	MK883411	MK883359	GQ402431	Colomba et al. (2011, 2015, this work)
	Palermo: Cefalú, La Rocca	38.0389, 14.0264			KR921891	MK883310	MK883406	MK883358	GQ402432	Colomba et al. (2011, 2015, this work)
	Palermo: Cefalú, La Rocca	38.0389, 14.0264			KR921892	MK883311	MK883394	MK883360	GQ402433	Colomba et al. (2011, 2015, this work)
*Erctella mazzullii*	Palermo: Monte Pellegrino	38.1633, 13.3569			KR921909	MK883323	MK883401	MK883353	GQ402449	Colomba et al. (2011, 2015, this work)
	Palermo: Monte Pellegrino	38.1633, 13.3569			KR921910	MK883324	MK883402	MK883374	GQ402450	Colomba et al. (2011, 2015, this work)
	Palermo: Monte Pellegrino	38.1633, 13.3569			KR921911	MK883325	MK883418	MK883354	GQ402451	Colomba et al. (2011, 2015, this work)
	Palermo: Cinisi, Monte Pecoraro	38.1578, 13.1283			KR921912	MK883319	MK883421	MK883365	GQ402454	Colomba et al. (2011, 2015, this work)
	Palermo: Cinisi, Monte Pecoraro	38.1578, 13.1283			KR921913	MK883320	MK883419	MK883366	GQ402455	Colomba et al. (2011, 2015, this work)
	Palermo: Cinisi, Monte Pecoraro	38.1578, 13.1283			KR921914	MK883321	MK883420	MK883367	GQ402456	Colomba et al. (2011, 2015, this work)
	Palermo: Sferracavallo	38.1953, 13.2719			KR921901	MK883318	MK883415	MK883369	GQ402435	Colomba et al. (2011, 2015, this work)
	Palermo: Sferracavallo	38.1953, 13.2719			KR921902	MK883312	MK883395	MK883370	GQ402436	Colomba et al. (2011, 2015, this work)
	Palermo: Sferracavallo	38.1953, 13.2719			KR921903	MK883317	MK883407	MK883364	GQ402437	Colomba et al. (2011, 2015, this work)
*Erctella mazzullii*	Palermo: Sferracavallo	38.1953, 13.2719			KR921904	MK883322	MK883416	MK883371	GQ402438	Colomba et al. (2011, 2015, this work)
	Palermo: Sferracavallo	38.1953, 13.2719			KR921905	MK883313	MK883417	MK883372	GQ402439	Colomba et al. (2011, 2015, this work)
	Palermo: Carini, Monte Columbrina	38.1583, 13.2292			KR921906	MK883314	MK883397	MK883373	GQ402444	Colomba et al. (2011, 2015, this work)

The PCR included the following admixture: 2 µL template, 12.5 µL GoTaq (Promega) polymerase, 8.5 µL of nuclease-free water and 1 µL of both forward and reverse primer (10 µmol) respectively. In cases where the PCR signal was judged too weak, the reaction was repeated using 3 µL template DNA, 3 µL of the previous PCR product and 5.5 µL of nuclease-free water. The amount of GoTaq and primers stayed the same. The PCR was conducted using the following protocols: For COI, the admixture was first heated up to 95 °C for 1 minute (min), followed by 30 cycles of 30 seconds (s) at 95 °C, 30s at 52 °C and 30s at 72 °C, finishing with 3 min at 72 °C. For 16S, the protocol started with 2:30 min at 90 °C, followed by ten cycles of 30s at 92 °C, 30s at 44 °C and 40s at 72 °C, followed again by 30s at 92 °C, 40s at 48 °C and 40s at 48 °C. The protocol for 28S started with 1 min at 96 °C, then went into 35 cycles of 30s at 94 °C, 30s at 50 °C and 1 min at 72 °C, finishing with 10 min at 72 °C. The ITS2 protocol started with 1 min at 96 °C, followed by 35 cycles of 30s at 94 °C, 30s at 44 °C and 1 min at 72 °C, ending with 10 min at 72 °C. For H3 the admixture was first heated up to 95 °C for 3 min, followed by 40 cycles of 45s at 94 °C, 45s at 50 °C and 2 min at 72 °C, finishing with 10 min at 72 °C. The protocols for COI and H3 could be used for both markers. The PCR products were sequenced at the LGC Genomics GmbH (Berlin, Germany) and at Eurofins Genomics (Ebersberg, Germany) using their respective standard protocol. In total, 48 helicid specimens were used, chiefly from the genera *Cantareus*, *Cornu*, and *Erctella*. Five specimens, belonging to *Helix pomatia*, *Massylaea vermiculata*, and *Massylaea constantina* were used as outgroup. Sequences received from LGC and Eurofins were imported into the Geneious 5.4.7 software (Kearse et al. 2012). The forward and reverse sequences for each gene and individual were combined and edited. For each marker, sequences were aligned in Geneious using the MAFFT multiple sequence alignment plugin version 1.3.6 (based on MAFFT v7.308; Katoh et al. 2002, Katoh and Standley 2013), letting the program choose the appropriate algorithm. The sequence length of each alignment was standardized to the length mentioned above. The alignments were concatenated using the “Concatenate sequences or alignments” function in Geneious.

Topologies were estimated using two different phylogenetic methods: Maximum Likelihood (ML) and Bayesian inference (BI). The five markers were set as partitions in both of these methods, using a distinct model for the third codon in protein-coding genes (COI, H3). The Maximum Likelihood (ML) topology was estimated using the RAxML 7.2.8 (Stamatakis 2014) plugin of Geneious with the GTR gamma Nucleotide model and 1’000 bootstrap replicates.

**Figure 1.**
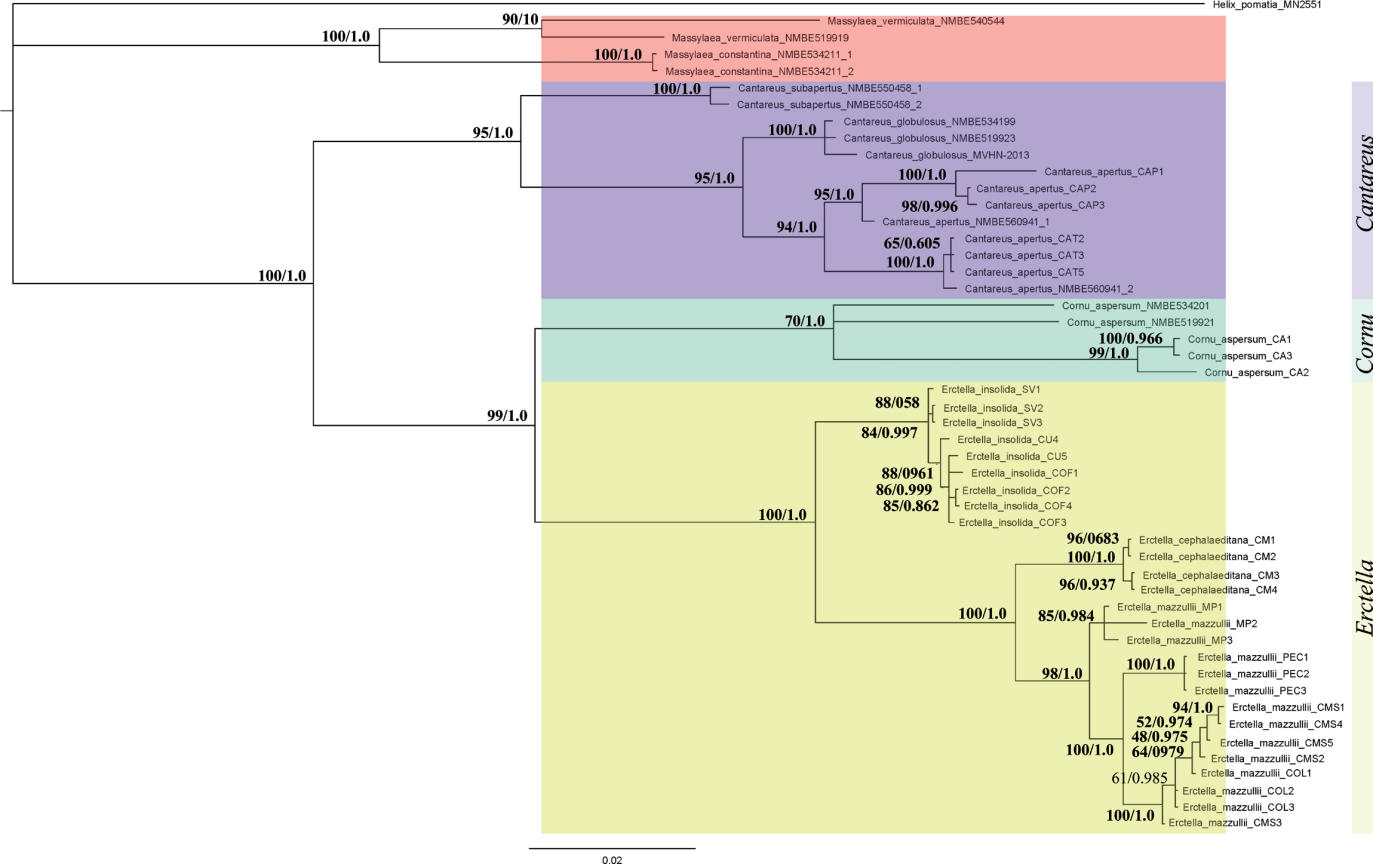
Combined RaxML and Bayesian tree of *Cantareus*, *Cornu*, and *Erctella*, using COI, 16S, H3, 28S, and ITS2.

The Bayesian tree, which was used as a basis for the combined tree (Fig. 1), was reconstructed with MrBayes 3.2.6 (Huelsenbeck and Ronquist 2001) using the mixed substitution model (which incorporates model testing into the MCMC), invgamma rate variation, a Markov Chain Monte Carlo (MCMC) chain length of 10,000,000 generations, a subsampling frequency of every 4,000 generations, the first 100,000 generations were discarded as burn-in, four heated chains and a chain temperature parameter of 0.2. Calculations were performed on the UBELIx (http://www.id.unibe.ch/hpc), the HPC cluster at the University of Bern.

#### Molecular taxonomy

The Bayesian and RaxML reconstructions yielded the same topology for all species involved and are shown in Fig. 1.

All three genera treated here in the analysis split in monophyletic lineages, and the nodes on the generic level have high support values. The species *H. subaperta* turned out to be a member of *Cantareus* rather than of *Cornu*, as could be expected by the colour pattern of its shell. The specimens from northern Africa, which had been identified as *C. apertus* so far, form a well-supported (95/1) lineage separate from all Italian specimens available in the study. For this species, the nominal taxon name Helix aperta var. globulosa Bourguignat, 1863 from Constantine is available. It should be stressed that the specimen MVHN_2013 (Razkin et al. 2015) originates from Djelfa, a city in the southwest of Tizi Ouzou (shell not seen by the present authors). The Italian specimens of *C. apertus* show some genetic differentiation as they split into two major clades; interestingly, the two specimens from Amantea in Calabria (NMBE 560941) occur each in one of these lineages. The addition of nuclear markers in *Erctella* consolidated their topology on species level with high support values.

**Table 2.** The five markers used in this study.

Marker	Primer Name	Primer sequence	Reference
COI	LCO1490	5’-GGTCAACAAATCATAAAGATATTGG-3’	Folmer et al. (1994)
	HCO2198	5’-TAAACTTCAGGGTGACCAAAAAATCA-3’	
16S	16s F	5’-CGGCCGCCTGTTTATCAAAAACAT-3’	Palumbi et al. (1991)
	16s R	5’-GGAGCTCCGGTTTGAACTCAGATC-3’	
28S	LSU-2	5’-GGGTTGTTTGGGAATGCAGC-3’	Wade and Mordan (2000)
	LSU-4	5’-GTTAGACTCCTTGGTCCGTC-3’	
5.8S-ITS2-28S	ITS2ModA	5’-GCTTGCGGAGAATTAATGTGAA-3’	Bouaziz-Yahiatene et al. (2017)
	ITS2ModB	5’-GGTACCTTGTTCGCTATCGGA-3’	
H3	H3-F	5’-ATGGCTCGTACCAAGCAGAC(ACG)GC-3’	Colgan et al. (1998)
	H3-R	5’-ATATCCTT(AG GGCAT(AG) AT(AG)GTG-3’	

**Table 3.** Character matrix including the genera Cantareus, Cornu, Erctella and Rossmaessleria. 1. Last whorl of the shell: 0: occupying more than two thirds of the shell height, 1: occupying less than two thirds of the shell height – 2. Teleoconch colour patterns: 0: none, 1: up to 5 spiral bands, 2: a reticulate pattern – 3. Teleoconch surface: 0: smooth, sometimes with longitudinal riblets and growth lines, 1: granulated, 2: with wrinkles, 3: strongly wrinkled and irregularly reticulated, 4: ribbed – 4. Penis form: 0: short, 1: elongate – 5. Epiphallus length: 0: as long as penis, 1: at least three times the length of penis – 6. Penial flagellum: 0: twice the length of the epiphallus, 1: clearly more than twice the length of the epiphallus – 7. Penial lumen: 0: with numerous crests; 1: smooth – 8. PP1 0: not shifted laterally, 1: shifted laterally, leaving a small pore as a connection between epiphallus and penis near its base – 9. PP2: 0: pp2 reduced to a septum, 1: reduced to a annular pad, 2: pp2 present – 10. Diverticulum: 0: as long as vesicle stem + vesicle, 1: slightly longer than vesicle stem + vesicle, 2: much longer (twice and more) than vesicle stem + vesicle, V: length variable – 11. Atrial stimulator: 0: small, 1: medium, 2: large.

	*Cantareus apertus*	*Cantareus koraegaelius*	*Cantareus subapertus*	*Cornu aspersum*	*Erctella insolida*	*Erctella mazzullii*	*Erctella cephalaeditana*	*Rossmaessleria scherzeri*
1	0	0	0	0	0	0	0	1
2	0	0	1	2	0	0/1	0	1
3	0	0	1	0	0	2	3	0/4
4	0	1	0	1	0	0	0	NA
5	0	0	1	0	0	0	0	0
6	0	0	0	1	0	0	0	1
7	0	1	1	NA	0	0	0	NA
8	1	1	1	1	1	1	1	0
9	0/2	2	2	1	1	1	1	2
10	1	2	1	0	0	2	1	V
11	1	2	2	2	0	0	0	0/1

## Taxonomic implications

### Cantareus

Taxon classificationAnimaliaStylommatophoraHelicidae

Risso, 1826

2E3171AA-AA64-539F-82DC-2DFCA74FE2D8

Cantareus Risso, 1826, Histoire naturelle des principales productions de l’Europe Méridionale, IV: 64.

#### Notes.

In Table 3, the most important character states of the shells and the genital organs of the Otalini subgroup according to Razkin et al. (2015: 108, Fig. 2) including the genera *Cantareus*, *Cornu*, and *Rossmaessleria* are shown. Within the Otalini, *Cantareus*, *Cornu*, and *Erctella* share the synapomorphy of a globular to slightly conical shell, other genera in the tribe tend to have more flattened shells (character 1). In all other shell traits, there is no apomorphy that discriminates between *Cantareus*, *Cornu*, and *Erctella* on generic level. On the level of the genital organs, the three genera share the synapomophy of the simple pore connecting epiphallus and penial chamber (character 8), while *Rossmaessleria* shows the plesiomophic state with two functional penial papillae. In *Cornu*, the flagellum is much longer than in the other genera. The phylogenetic value of this character state is not clear within the Otalini, within the Helicini, it is considered a plesiomorphy (Neubert 2014). Other character states like ratios in the bursa copulatrix complex (character 10). A massive atrial stimulator can be found in *Cantareus* and *Cornu*, while in *Erctella*, it is relatively small (character 11). Large and massive stimulators are found in many taxa of Helicidae, so a reduction of this system is here interpreted as an apomorphic character state.

**Figure 2.**
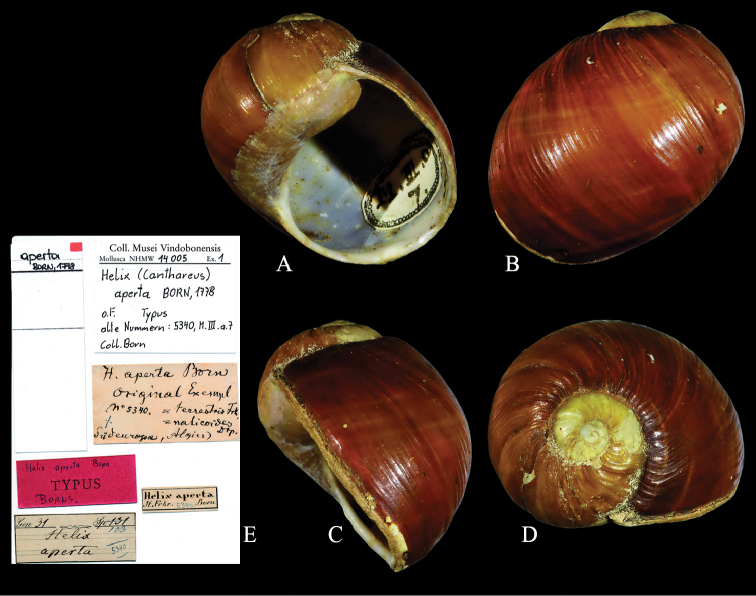
*Cantareus apertus*. Syntype of *Helix aperta*NHMW-MO 14005, shell diameter 28.75 mm. Shell in **A** frontal **B** dorsal **C** lateral and **D** apical views **E** labels of the syntype lot. Photographs NHMW, × 1.5.

**Remarks.** The change of the status of *Cantareus* from a monotypic to a polytypic genus causes some nomenclatorial problems. The type species of the genus is *Helix naticoides* Draparnaud, 1801 from France, which so far has been considered a synonym of *Helix aperta* Born, 1778, with the specimen preserved in the Born collection in the NHMW as the name bearing syntype of *aperta* (Fig. 2).The origin of Born’s specimen is unknown, and there are almost no shell morphological differences to the Algerian lineage, which proved to represent a separate species (Fig. 1). The correct origin of Born’s specimen could probably be clarified genetically by applying NGS methods using shell fragments of the syntype specimen, but this is beyond the scope of this paper.

Anticipating a north African origin of the syntype NHMW-MO 14005 by fixing its type locality in Algeria ends up in a chaotic rearrangement of species names in the group. For Europe, the name *naticoides* would be reactivated with its last use as an accepted species in 1850 (!). The north African species would then be named *apertus* contradicting 170 years of permanent use. By fixing the use of the name *Helix koraegaelia* Bourguignat in Locard, 1882, to the north African lineage, this problem is resolved, and the stability or universality of names used in zoology is guaranteed.

*Cantareus apertus* is well known for its protective behaviour, which gave the genus its name “*Cantareus*: the singer”. Once disturbed (Fig. 23), the species is able to press the air in its lung cavity through the pneumostome producing a series of tweeking sounds (https://youtu.be/CWOhZWLkd4o).

### Cantareus apertus

Taxon classificationAnimaliaStylommatophoraHelicidae

(Born, 1778)

CDBF9789-D4DD-5E82-BCEF-F219E58E6C08

**Figure 3.**
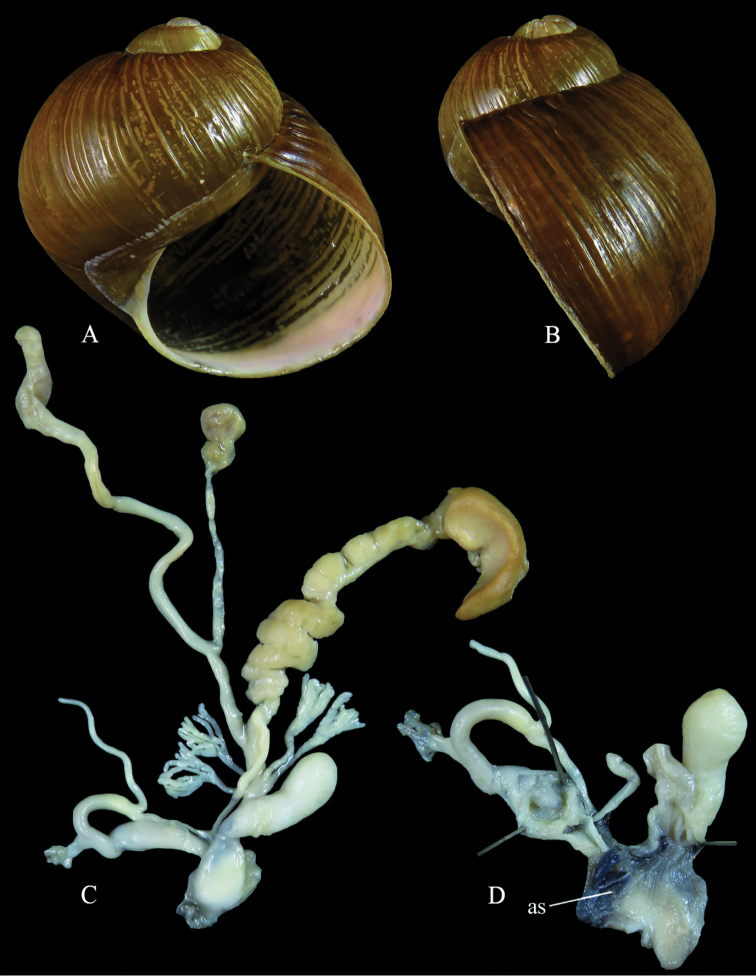
*Cantareus apertus*. Italy, Foggia, Ordona **A** shell in frontal (left) and **B** lateral (right) view; genital anatomy: situs (**C**) and a section showing the male genital tract and the atrium (**D**). Photographs F. Liberto.

**Figure 4.**
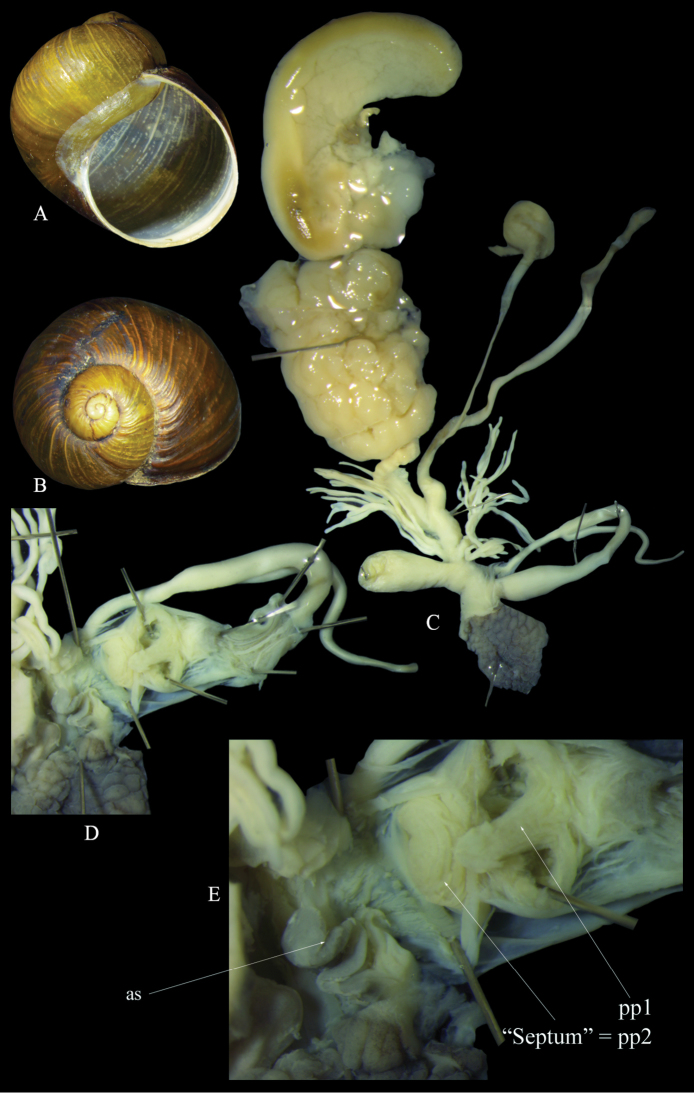
*Cantareus apertus*. Italy, Calabria, Amantea, Marincola, NMBE 560941 **A** shell in frontal and **B** lateral views **C** genital anatomy, situs **D, E** details showing the male genital tract and the atrium. Photographs E. Neubert, shell × 1.5.

Figs 2 , 3 , 4 , 21–24

Helix aperta Born, 1778, Index rerum naturalium Musei Caesarei Vindobonensis, I. Testacea: 399 [no type locality mentioned].
Helix naticoides : 1801, Draparnaud, Tableau des mollusques terrestres et fluviatiles de la France: 78–79 [France, la Provence, à Antibes, à Cannes].

#### Type material.

Syntype *aperta*: NHMW-MO 14005.

#### Specimens examined.

Italy: Foggia, Ordona, 41.313889N, 15.622222E, 12.10.2018, leg. G. Martucci (ex coll. Sparacio 5031/9), coll. Liberto (Fig. 3); Calabria, Amantea, Marincola, 39.112778N, 16.079722E, 7.10.2018, leg. W. Renda, NMBE 560941/2 (preserved), ex coll. Liberto (Fig. 4).

#### Description.

Shell thick, medium sized if compared to other helicid species, with a depressed spire and a large last whorl occupying more than two thirds of the complete height of the shell; protoconch small, consisting of 1.5 smooth whorls; teleoconch consisting of approximately 4 whorls, separated by a deep, sometimes crenulated suture; basic colour of teleoconch greenish-brownish, often with longitudinal yellow streaks and a few scattered zig-zag markings; surface of teleoconch smooth, but also often covered by low longitudinal riblets; aperture almost perfectly rounded, enormously large, old specimens with an inconspicuous whitish lip; umbilicus always completely closed.

Genital organs: penis short, club-shaped, epiphallus short, of the same length as penis, mrp attaching in the distal third of epiphallus or even closer to penis; flagellum twice the length of the epiphallus; atrial and penial lumen with numerous crests, penial chamber lumen is wrinkled, pp2 a short broad papilla with a central perforation structured by thick annuli to almost completely reduced forming a septum; pp1 a blind papilla, in a central position inside the penial chamber, elongate, sometimes with a broadened tip; epiphallial pore in a lateral position; distal epiphallial lumen with six broad pilasters, the proximal lumen with elongated ridges.

Vagina short, stem of pedunculus thickened and short, diverticulum slightly longer than the vesicle stem + vesicle, longer than the flagellum; glandulae mucosae longer than the dart sac, with a thickened basal part and two subsequent ramifications, tubules thin and weak, less than 10 tubules per stem; atrium with a medium sized stimulator flap.

#### Measurements.

Syntype NHMW: H = 28.25 mm; D = 28.75 mm; PH = 22.3 mm; PD = 19.2 mm.

#### Distribution.

South-eastern France including Corsica, Italy, Sicily, south-eastern Adriatic coast, Albania, western Greece; scattered found introduced on some Aegean Islands, and in Turkey, Muğla, Gökçebel (Örstan et al. 2005: 7).

#### Remarks.

The anatomy of the genital organs of *C. apertus* has been investigated by several authors, for example Hesse (1919), Germain (1930) and Giusti et al. (1995). Schileyko (2006: 1801, fi.g. 2308) presented also details of the penial lumina. In his picture of the genital organs of an animal collected in the surroundings of Pisa, Italy, he misinterpreted the morphology of the epiphallial papilla (pp1) suggesting that it was a functional papilla as in many other helicid genera (the illustrated shell comes from Arles, France, and thus does not belong to the dissected specimen). Secondly, in his specimen, the penial papilla (pp2) was completely reduced, so only the perpendicular wall forming the basis of pp2 was left. This led to the misapprehension that in the genus *Cantareus*, this papilla is reduced, and only a “septum” is left in the place of the papilla.

### Cantareus subapertus

Taxon classificationAnimaliaStylommatophoraHelicidae

(Ancey, 1893)

505669E9-0D78-5DD3-80CF-4DABE604E75D

Figs 5–8 , 10–14 , 25

Helix subaperta : Ancey 1893, Bulletin de la Société Zoologique de France 18(3): 136–138 [la chaîne du Djurdjura, en Kabylie; published 20 June 1893].
Helix mazzuliopsis : Ancey 1893, Bulletin de la Société Zoologique de France 18(3): 136 [name mentioned in footnote; not an available name according to Article 11.6.1 as it has been published as a synonym and has not been treated as an available name before 1961].
Helix mazzulopsis : 1893, Pilsbry, Manual of Conchology (2)8(32): 238, pl. 46, figs 41, 42 [Jurjura Mts., Algeria; published 1 July 1893; lectotype designation by Baker (1963: 258)].

#### Type specimens.

*Mazzulopsis*: lectotype ANSP 63133, paralectotype ANSP 459220. *subaperta*: 3 syntypes, NHMW 7861, NHMW 7862, NHMW 7863; paratypes SMF 75256/8, coll. Nägele ex Ancey, the original label of Ancey contains the additional information “Dra-el-Mizan, 1893”.

#### Specimens examined.

Algeria, Kabylie: Tiguemounine (Ouacif), 1100 m alt. coll. Bouaziz; Ighil Bourmi (Ait Bouaddou), 950 m alt. NMBE 550458; ditto, le. F. Medjoub, NMBE 555649; Ait Houari (Assi Youcef), 1000 m alt. coll. Bouaziz; Tizi Guefres (Iferhounene), 1100 m alt coll. Bouaziz. The Senckenberg Research Institute houses > 30 shells of this species, all of them from “Kabylie” and/or “Djudjura”.

#### Description.

Shell medium sized to large, thin, globose with a broad to relatively acute conical spire; protoconch whitish, large, with a diameter of up to 6 mm and 2.5 smooth whorls; basic shell colour olive yellowish with up to five separate brown spiral bands; teleoconch covered by a dense granulation, sometimes accompanied by very fine, deep spirals; teleoconch usually covered by irregularly arranged riblets of even ribs, usually stronger around the umbilical area; periostracum thick, often preserved on the shell in small patches; in eroded shells, ribs and riblets whitish; aperture large, elongate oval, slightly thickened forming a lip callus, with a parietal callus in fully adult specimens; aperture whitish inside, with the spiral bands shining through the thin shell; peristome sharp; umbilicus closed, periomphalum covered by a thickened calcareous layer.

**Figures 5–9.**
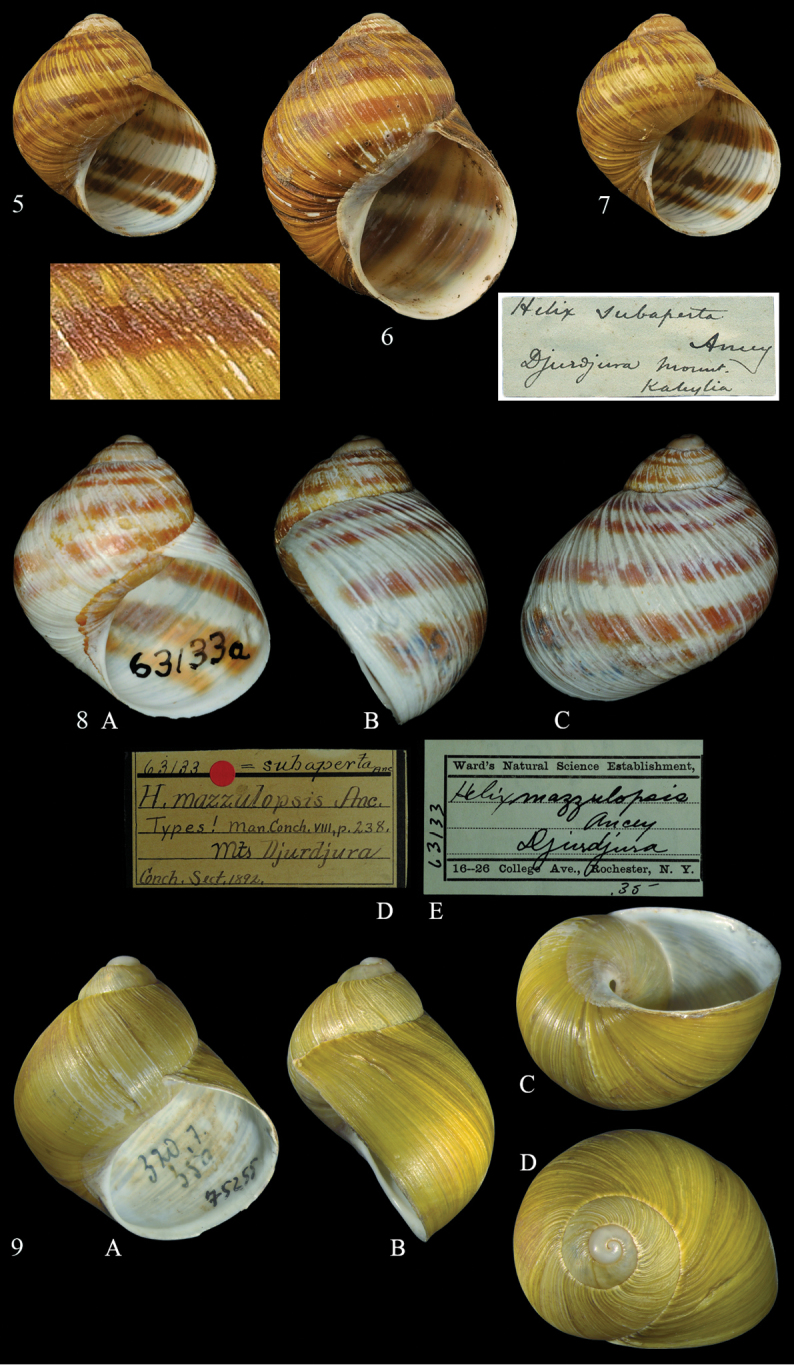
*Cantareus subapertus* (Ancey, 1893). **5–7** syntypes *Helix subaperta*NHMW, Djurdjura, Kabylie ex Ancey **5**NHMW 7861, D = 23.44 mm **6**NHMW 7862, D = 23.51 mm **7**NHMW 7863, D = 29.44 mm **8***Helix mazzulopsis* lectotype ANSP 63133, Jurjura Mts. Shell in frontal (**A**) lateral (**B**) and dorsal (**C**) view (**D, E**) labels **9** “*Helix aspersa*“, original specimen of Iconographie (2) 3, pl. 69, fig. 359. Shell in frontal (**A**) lateral (**B**) ventral (**C**) and apical (**D**) views. Photographers **5–7** H. Wood, NHMW; photograph **8** E. Wildner, ANSP; photograph **9** E. Bochud, NMBE; all shells × 1.5.

#### Genital organs.

Penis short, epiphallus reaching at least three times the length of penis, mrp attaching in the distal third of epiphallus; flagellum twice the length of the epiphallus; penial lumen smooth, pp2 a short broad papilla with a central perforation structured by thick annuli, pp1 a blind papilla, the epiphallial pore in a lateral position; distal epiphallial lumen with broad pilasters, the proximal lumen with elongated ridges.

Vagina short, stem of pedunculus thickened and short, diverticulum longer than the vesicle stem + vesicle, longer than the flagellum; glandulae mucosae longer than the dart sac, with a thickened basal part and two subsequent ramifications, tubules thin and weak, less than 10 tubules per stem; atrium dominated by a massive stimulator.

#### Measurements.

Syntypes figured (n = 4): H = 26 mm; D = 27.5 mm; PH = 17.7 mm; PD = 15.4 mm.

#### Distribution.

As far as known, this species is restricted to the Djudjura Mts., where it inhabits quite high altitudes. It also occurs in the northern promontory of this mountain ridge.

#### Remarks.

In the description of *Helix subaperta*, Ancey (1893) mentioned in a footnote that he already shared this species under the name *H. mazzuliopsis* with his correspondents. Pilsbry’s shells (1893) were purchased from a shell dealer (see label in Fig. 8E) bearing the name *H. mazzulopsis*, which he consequently used! Moreover, Pilsbry remarks: “I have been unable to find any description or mention of this form in the literature”; thus, *H. mazzulopsis* cannot be considered an emendation or an incorrect subsequent spelling of *H. mazzuliopsis*. Strictly speaking, he introduced a new name, and corrected his error two years later in the “Index to Helices” (Pilsbry 1895: 120) with the note “For *H. mazzulopsis* read *H. subaperta*. Ancey’s description appeared June 20; that in Man. Conch., July 1”.

### Cantareus koraegaelius

Taxon classificationAnimaliaStylommatophoraHelicidae

Bourguignat in Locard, 1882

D577F653-270F-5183-8B0F-06B1D86C7A8C

Figs 15 , 16–20

Helix aperta var. globulosa : Bourguignat 1863, Malacologie de l’Algérie, I: 96, pl. VII, figs 3 & 4 [environs de Constantine] [non Helix (Helicogena) globulosa A. Férussac, 1821, Tableau systématique de la famille des Limaçons, livr. 10: 28 (Quarto edition; Folio edition = page 32) (published 26 May 1821). There is no description but refers to plate 25, figs 3 & 4; this plate was published in livraison 5 (4 December 1819) nec Helix globulosa von Zieten, 1832, Die Versteinerungen Württembergs Heft 5: 38, pl. 29, fig. 3a-c].
Helix koraegaelia : Bourguignat in Locard 1882, Prodrome de malacologie française. [I]. Catalogue général des Mollusques vivants de France. Mollusques terrestres, des eaux douces et des eaux saumâtres 51: 302 [la Provence au nord, et le Sahara au sud jusqu’à l’Asie-mineure, embrassant la Corse, la Sardaigne, La Sicile, l’Italie, les îles Ioniennes, la Grèce et les îles de l’Archipel].

#### Type material.

*Globulosa*: lost. *koraegaelia*: lectotype [sic!] MHNG-MOLL 117907 from Algeria; type locality: “Djemaa N’Saharidj” (= Djama-N-Saharidj) [Djemaa Saharidj, Mekla, 36.683484° 4.288257°].

#### Remarks.

*Cantareus koraegaelius* is a species that is almost inseparable from its congener *C. apertus*. This also explains why Bourguignat recorded this species from the complete distribution area of the latter species (and including the Algerian lineage). All specimens left in Bourguignat’s collection originating from the localities mentioned are syntypes of *Helix koraegaelia*. Thus, the type lots contain two different species. To unambiguously fix the use of this specific name, we herewith select the single specimen MHNG-MOLL 117907 from “Djemaa N’Saharidj” in Algeria as lectotype. This locality in Tizi Ouzou is very close to the places, where the anatomically and genetically well-known specimens (see below) have been recorded. The application of the name *H. koraegaelia* is herewith restricted to specimens exhibiting the character states as explained in this paper forming a new species.

#### Additional specimen examined.

Algeria: Tigzirt/ Tizi Ouzou/ Kabylie, NMBE 534199/1 (specimen preserved and sequenced); Draa Ben Khedaa/ Tizi Ouzou/ Kabylie, NMBE 519923/1 (preserved and sequenced specimen).

#### Description.

Shell thin, medium sized, with a relatively elevated spire and a large last whorl occupying more than half of the complete height of the shell; protoconch medium-sized, consisting of 1.5 smooth whorls; teleoconch with approximately four whorls, separated by a deep, crenulated suture; colour of teleoconch brownish, surface of teleoconch covered by low longitudinal riblets, which are more prominent below the suture, disappearing on the last whorl; aperture rounded, very large, with an inconspicuous whitish lip; umbilicus always completely closed.

#### Genital organs.

penis elongate, club-shaped, epiphallus as long as penis, mrp attaching in the distal third of epiphallus or even closer to penis; flagellum twice the length of the epiphallus; penial lumen smooth; pp2 a broad acute conical papilla with a central perforation structured by thick annuli; pp1 a blind papilla with a slightly broadened apex, the epiphallial pore in a lateral position; atrial and penial lumen with numerous strong crests; distal epiphallial lumen with six broad pilasters, the proximal lumen with elongated ridges.

Vagina short, stem of pedunculus thickened and short, diverticulum extremely longer than the vesicle stem + vesicle, and almost three times longer than the flagellum; glandulae mucosae longer than the dart sac, with a thickened basal part and two subsequent ramifications, tubules thin and weak, less than 10 tubules per stem; atrium dominated by a massive stimulator flap.

#### Measurements

(of lectotype): H = 26.3 mm; D = 27.0 mm; PH = 21.8 mm; PD = 17.7 mm.

#### Distribution.

the two genetically identified specimens originate from Eastern Algeria.

#### Remarks.

The description of the genital organs is based on the specimen NMBE 534199 from Tigzirt; unfortunately, the other specimen from Draa Ben Khedaa was subadult with only partially developed genital organs.

It is almost impossible to define differences in shell morphology between this new species and *C. apertus*. In the two genetically identified specimens, the protoconch of *C. koraegaelius* seems to be larger than in *C. apertus*, and the shell colour is more or less uniformly brown without any yellowish or greenish streaks. However, the morphology of the genital organs is in fact different: the large triangular pp2 is strikingly different to all what is known so far from the Italian *C. apertus*, where pp2 is very short to almost completely reduced, so that a “septum” is left.

### Cantareus

Taxon classificationAnimaliaStylommatophoraHelicidae

species (?)

1DD04E04-ED07-5552-A529-586A163AE9FF

Fig. 9

Helix aspersa : Kobelt 1888, Iconographie (2) 3: 9–10, pl. 69, figs 359 & 360 [non Helix aspersa O. F. Müller, 1774 [Gorges d’Isser bei Palestro].
Helix (Cryptomphalus) aspersa : Sacchi 1955, Italian Journal of Zoology 22(2): 638, pl. 3, fig. E [Palaestro].

This specimen was collected by Kobelt in the Gorge d’Isser; it lacks the malleation typical for *Cornu aspersum*, and thus is here considered to rather constitute a species in *Cantareus* than in *Cornu*. However, it also lacks the riblets on the teleoconch, but also has the typical granulation on the whorl exactly like in the specimens from the Djudjura Mts. This form might represent another species close to *C. subapertus*, but preserved specimens from the canyon are needed to decide about its status. This form might be a separate species endemic to the Gorge d’Isser.

**Figures 10–14.**
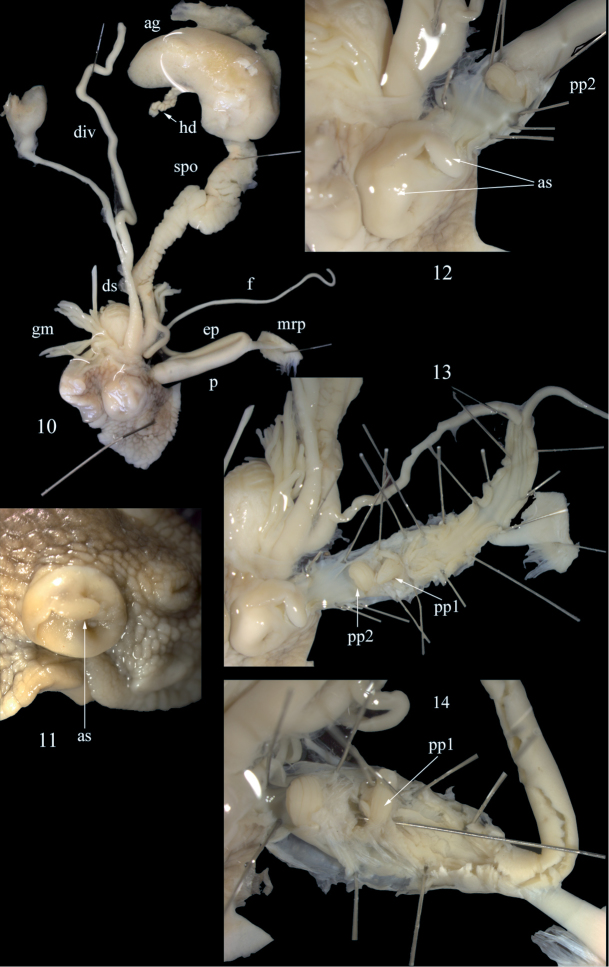
*Cantareus subapertus*. Anatomical details of the genital organs; specimen collected at Ighil Bourmi, leg. H. Bouaziz- Yahiatene, NMBE 550458 **10** situs of the genital organs, 46 mm total length **11** partly everted genital atrium with the atrial stimulator **12** distal penial tube with pp2 **13** penis and epiphallus completely opened showing both papillae, and the internal structure of the penial chamber and the epiphallus **14** detail of the penial lumen; note: the needle represents the epiphallial canal, with pp2 bent upwards to show the ending of the canal. Abbreviations: ag = albumen gland; as = atrial stimulator; div = diverticulum; ds = dart sac; ep = epiphallus; f = flagellum; gm = glandulae mucosae; hd = hermaphroditic duct; mrp = musculus retractor penis; p = penis; pp1 = penial papilla 1; pp2 = penial papilla 2; spo = spermoviduct. All figures not to scale.

**Figure 15.**
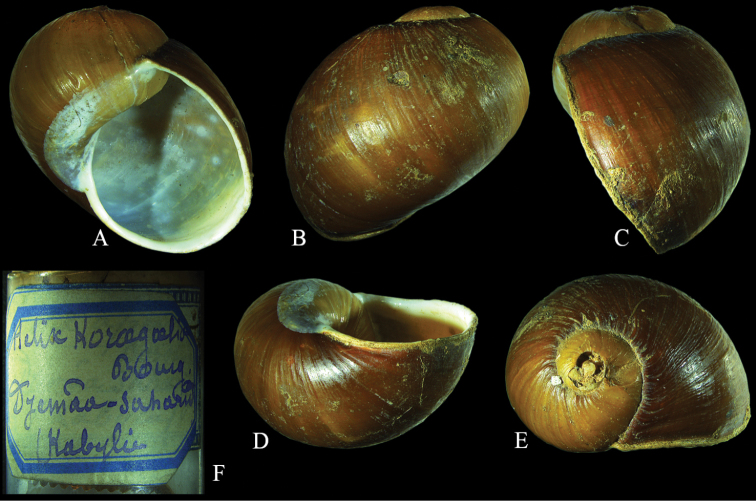
*Cantareus koraegaelius*. Lectotype of *Helix koraegaelia* MHNG-MOLL 117907, shell diameter 27.0 mm. Shell in **A** frontal **B** dorsal **C** lateral **D** ventral and **E** apical views **F** label. Photographs T. Inäbnit, NMBE, × 1.5.

**Figures 16–20.**
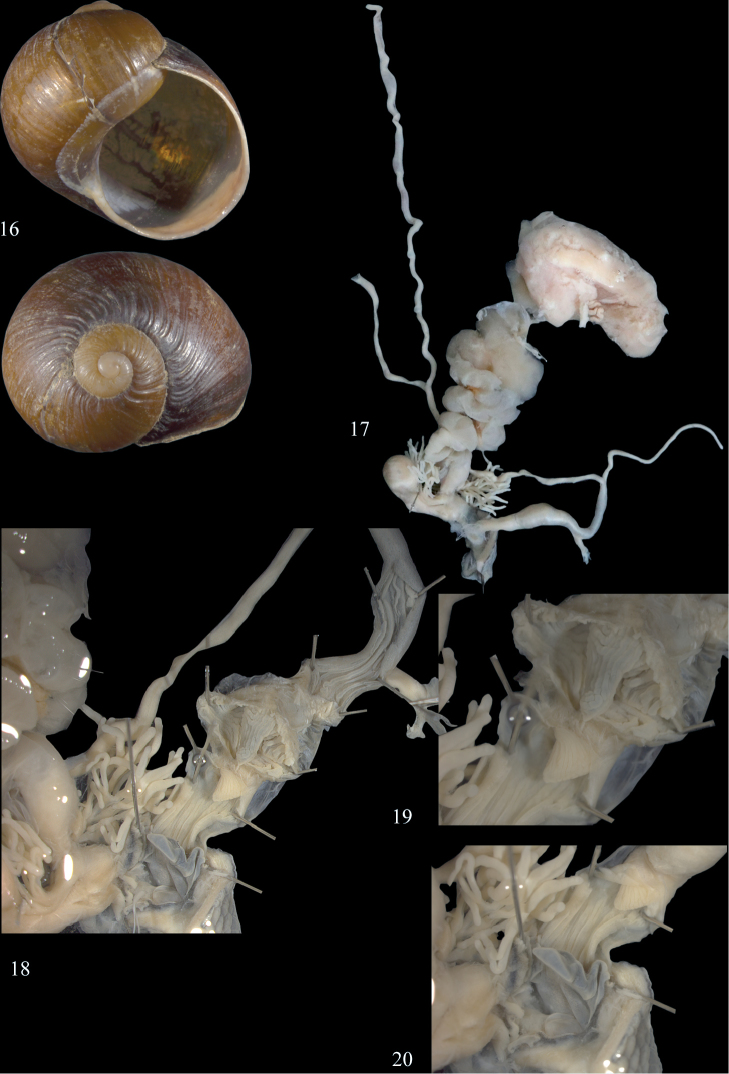
*Cantareus koraegaelius.* Shell and anatomical details of the genital organs of dissected and sequenced specimen NMBE 534199; specimen collected at Tigzirt, Tizi Ouzou, Kabylie **16** shell; shell diameter 28.1 mm **17** situs; situs length 57.5 mm **18** lumina of epiphallus, penial chamber, penial papillae and atrium **19** penial papillae **20** atrium with atrial stimulator. Photographs E. Neubert, shell × 1.5.

**Figures 21–25.**
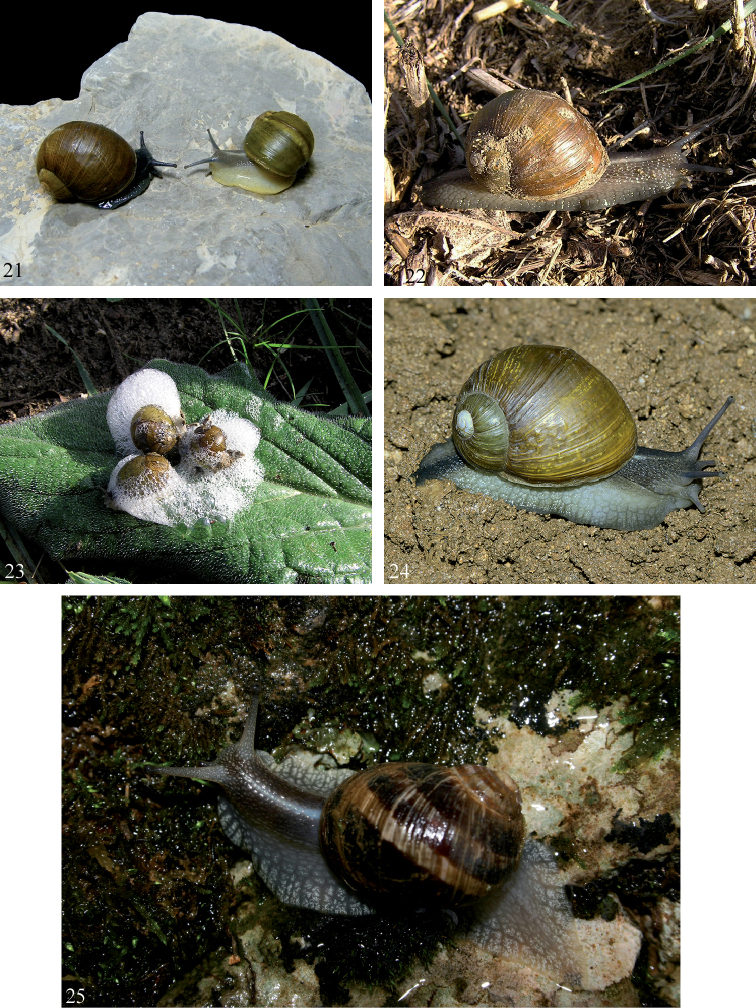
Pictures of living specimens of *Cantareus* species **21***Cantareus apertus*: on the left a black specimen with brown shell from Roccapalumba, Sicily, Italy, 15.XI.2009 (Coll. F. Liberto 5532); on the right a yellow specimen with green shell from Prizzi, Sicily, Italy, 15.XI.2009 (Coll. F. Liberto 5545) **22***Cantareus apertus*, Niscemi, Sicily Italy, 22.X.2016 **23***Cantareus apertus*, Niscemi, Sicily, Italy, 22.X.2016, specimens defending themselves by emitting bubbles of slime and a series of tweeting sounds **24***Cantareus apertus*, Foggia, Ordona, 41.313889N, 15.622222E, 12.10.2018, leg. G. Martucci (photo/collection I. Sparacio 5031/9) **25***Cantareus subapertus*, Algeria, Parc National du Djudjura, 1700 m, 11.X.2008 (Photographs Vela Errol).

## Discussion

The main results of this work consist of the allocation of *H. subaperta* in the genus *Cantareus*, and the recovery of a third species in *Cantareus*, i.e., *C. koraegaelius*. The minute granulation of the teleoconch, which is a new shell morphological trait for *Cantareus*, can also be found in other Helicidae like for example *Helix* Linnaeus, 1758 (Neubert 2014), and thus represents a plesiomorphic character state above the species level.

*Cantareus koraegaelius* can almost be considered a cryptic species, because its shell does not deviate in any major trait from the shells of its sibling species, *C. apertus* (Born, 1778). The separation between *C. apertus* and *C. koraegaelius* is mainly based on the clear genetic data, and all traits discussed to separate the shells of the two species have currently to be considered as first impressions. Only the shell morphological and anatomical study of a larger number of specimens from the Algerian clade can corroborate the stability of the characters discussed here. It also has to be proven whether or not *C. apertus* is also present in Algeria, which might well be possible. The distance between Tizi Ouzou and Constantine is > 200 km as the bird flies, so it can be estimated that *C. koraegaelius* constitutes a more widespread species than *C. subapertus*, which in fact seems to be a small-range endemic species restricted to submontane to alpine environments of the Djudjura Moutains.

The data presented here suggest the need for a more careful investigation of the phylogenetic relationships among the populations of *C. apertus* from Sicily and southern Italy. Recent studies on species with a wide Mediterranean distribution like *Rumina decollata* (Linnaeus, 1758), *Massylaea vermiculata* (O. F. Müller, 1774) and *Cornu aspersum* (O. F. Müller, 1774), have shown a remarkable genetic divergence (Prévot et al. 2013; Bouaziz-Yahiatene et al. 2017; Sherpa et al. 2018). This will also hold true for *C. apertus*, which is probably introduced by human activities to other Mediterranean areas like southern France and Greece.

Neubert and Bank (2006: 105) argue that the transformation of the papilla system represents a synapomophic character state for an “*Eobania* group” based on the state of knowledge of this time. Walther et al. (2016: 399) remark that this is wrong because *Massylaea* [= *Eobania*] is found on a cluster separate to the *Rossmaessleria*/*Cornu*/*Cantareus* lineage (Razkin et al. 2015: 108, fig. 2). Consequently, this transformation occurred convergently within Otalini. We fully concur with this statement, although we must note that Razkin et al. (2015: 114) also states “...Otalini in the concatenated-gene tree and Helicini in the nuclear rRNA tree were not supported by NJ analysis”. The current research on the Otalini will hopefully include the missing genera, and deliver enough data to stabilise the phylogenetic structure of the tribe.

## Supplementary Material

XML Treatment for Cantareus

XML Treatment for Cantareus apertus

XML Treatment for Cantareus subapertus

XML Treatment for Cantareus koraegaelius

XML Treatment for Cantareus
